# Letter to Editor

**Published:** 2013

**Authors:** Amosy E. M’Koma

**Affiliations:** School of Medicine, Meharry Medical College, Nashville, USA: amkoma@mmc.edu

*Advances in Enzyme Research* (AER) *is pleased* to bring you multiple new topical collections on enzyme sciences. AER, a broad-based journal, was founded on tenets to publish the most exciting researches with enzyme science advances and to encourage research, leadership and application of enzymology in disease and provide a rapid turn-around time possible for reviewing and publishing and to disseminate the articles freely for research, teaching and reference purposes. As new Scientific Research Open Access model, AER publishes online peer-reviewed articles covering a wide range of the advancement of science and technology in academic disciplines. The behaviors of these enzymes as biological chemical in health and in disease, during and after medical and/or surgical interventions make AER journal a center of academic discipline in science. Enzymes are “biological catalysts” produced or derived from living organism. When an enzyme is formed, it is made by stringing together hundreds of amino acids in a very specific and unique order of chains and folds into a unique shape that allows the enzyme to carry out specific chemical reactions. An enzyme acts/speeds efficiently as a catalyst for a specific chemical reaction. The purpose of an enzyme in a cell is to allow the cell to carry out chemical reactions. These reactions allow the cell to build things or take things accordingly as needed. This is how a cell grows and reproduces. At the most basic level, a cell is really a little bag full of chemical reactions that are made possible by enzymes. At any given moment, all of the work being done inside any cell is being done by enzymes. If you understand enzymes, then you understand cells. A cell has about 1000 different types of enzymes floating around in the cytoplasm at any given time waiting for the chemical they recognize to float by. There are hundreds or millions of copies of each different type of enzyme, depending on how important a reaction is to a cell and how often the reaction is needed. These enzymes do everything from breaking glucose down for energy to building cell walls, constructing new enzymes and allowing the cell to reproduce. Enzymes do all of the work inside cells.

A good example is that you may have heard of people who are lactose intolerant. The problem arises because the sugar in milk, lactose, does not get broken into its glucose components [1]. Therefore, it cannot be digested. As the intestinal cells of individuals who are lactose intolerant do not produce enough lactase, the enzyme needed to break down lactose. This problem shows how the lack of just one enzyme in the human body can lead to significant problems. Sometimes there are medical solutions to such deficiencies but many enzyme deficiencies are not so easy to manage. A person who is lactose intolerant can swallow a drop of lactase prior to drinking milk or taking milk products and the problem is solved.

Further, there are diseases that can cause enzyme deficiency/malabsorption due to a number of gastrointestinal (GI) conditions such as: in atrophic gastritis, which increases with age, impairs the production of enzymes needed to break down food and also the production of intrinsic factor. Malabsorption would also occur with pancreatic insufficiency [2] and of course any surgery which removed part of e.g. the stomach [3] or small bowel [4-6] would increase risk. Intestinal conditions such as inflammatory bowel disease (Crohn’s disease (CD) and ulcerative colitis (UC) and celiac disease (COD) can cause significant problems. Long-term use of acid suppressants such as proton pump inhibitors, H_2_ antagonists is a potential risk factor, and these are some of the most widely prescribed and used drugs in the elderly population. Finally, in true pernicious anemia where there is an autoimmune component, there are three different types of antibodies that could be the cause. Those which bind to the intrinsic factor-vitamin B_12_ complex preventing absorption, antibodies which bind to intrinsic factor itself may prevent binding with cobalamin, and antibodies to gastric parietal cells preventing the production of intrinsic factor [7]. This type of enzyme sequential dependence act is essential physiology to understand in evaluating performance improvement in the treatment of disease in clinical medicine as well as in surgical practice as a whole. The biopatholphysiology of enzymes in different disease conditions is *the aim and scope of AER journal*. Review processing is performed by the Editorial Board Members of AER Journal who are *established* professionals in academia. We strongly believe that, removing barriers to research published online will greatly aid to the progress in AER scientific and technical disciplines. We hope you will find these new collections helpful and stimulating, and encourage you to provide us with your own suggestions of excellent articles for this and other future collections. Welcome.
